# Continuous serratus–intercostal plane block versus intercostal cryoanalgesia for postoperative pain after minimally invasive cardiac surgery: a prospective comparative observational study

**DOI:** 10.1186/s12871-026-03936-3

**Published:** 2026-05-23

**Authors:** Susana González-Suárez, Remedios Ríos-Barrera, Patricia Bascuñana Fornells, Berta Caralt Ramisa, Carlota M. Vigil-Escalera, Cherif M. Traore Kone, Isuru M. Dammala Liyanage, Martha Magaly Paguay Fernández, Rafael Rodríguez-Lecoq

**Affiliations:** 1 Department of Surgery, Universitat Autònoma de Barcelona, Unitat Docent Vall d’Hebron, Pg, de la Vall d’Hebron, 119-129, Barcelona, 08035 Spain; 2 Department of Anesthesiology, Hospital Universitari Vall d’Hebron, Barcelona, Spain; 3 Cardiovascular Diseases Research Group, Vall d’Hebron Institut de Recerca (VHIR), Barcelona, Spain; 4 Department of Cardiac Surgery, Hospital Universitari Vall d’Hebron, Barcelona, Spain; 5 Department of Intensive Care, Hospital Universitari Vall d’Hebron, Barcelona, Spain

**Keywords:** Thoracotomy, Postoperative pain, Regional anesthesia, Serratus-intercostal plane block, Intercostal cryoanalgesia

## Abstract

**Background:**

Minimally invasive cardiac surgery via minithoracotomy is associated with postoperative pain. Continuous BRILMA (block of the lateral branches of the intercostal nerves in the mid-axillary line) and intercostal cryoanalgesia are opioid-sparing techniques, but comparative evidence in this setting is limited.

**Methods:**

In this prospective comparative observational study, consecutive adults undergoing minimally invasive cardiac surgery received either continuous BRILMA or intercostal cryoanalgesia according to routine institutional practice. Treatment allocation was not determined by the study protocol and no randomization was performed. Pain at rest and during deep inspiration was assessed using the Numerical Rating Scale (NRS) after extubation and at 24, 48, and 72 h, discharge, and 30 days. The primary endpoint was pain at rest over the first 72 h. Secondary outcomes included inspiratory pain, opioid consumption during the first 72 postoperative hours, neuropathic pain, pulmonary complications, and hospital stay. Longitudinal analyses were performed using linear mixed models adjusted for age, sex, and surgical time.

**Results:**

Sixty-three patients were analyzed (BRILMA *n* = 33; cryoanalgesia *n* = 30). Pain decreased significantly over time in both groups (*p* < 0.001), with significant group-by-time interactions at rest (*p* = 0.002) and during deep inspiration (*p* < 0.001). BRILMA was associated with lower early pain scores, particularly at 72 h, whereas cryoanalgesia showed comparable or lower pain levels at 30 days. Although a greater proportion of patients in the cryoanalgesia group required rescue opioids, cumulative opioid exposure in the first 72 h was low in both groups. Neuropathic pain incidence was low and similar between techniques.

**Conclusion:**

BRILMA was associated with lower early postoperative pain scores, while cryoanalgesia showed a trend toward lower pain levels at 30 days. Overall opioid requirements and neuropathic pain rates were low, suggesting that both regional techniques may represent suitable regional analgesic strategies in this setting.

**Trial registration:**

The study was prospectively registered at ClinicalTrials.gov (NCT06086535; October 17, 2023). Ethical approval was obtained from the Clinical Research Ethics Committee of Vall d’Hebron University Hospital (Barcelona, Spain) (protocol number: PR(AG)324/2022; approved on November 25, 2022).

## Introduction

Over the past decade, cardiac surgery has progressively shifted toward less invasive approaches, with thoracotomy-based approaches emerging as safe and effective alternatives to conventional median sternotomy [1, 2]. This approach reduces surgical trauma and facilitates faster recovery [2, 3], but is frequently associated with significant acute postoperative pain due to muscle incision and rib retraction. Such pain may impair respiratory function, limit early mobilization and physiotherapy, and increase pulmonary complications [4–7].

Selecting an optimal analgesic strategy is therefore essential. In non-cardiac thoracic surgery, regional techniques have been widely studied. Previous studies have compared single- versus multilevel intercostal blocks [8], intercostal versus paravertebral or epidural techniques [9], and regional versus conventional systemic analgesia [10]. Intercostal cryoanalgesia has also been evaluated against conventional analgesia [11–14] and within multimodal analgesic regimens including paravertebral blockade [15].

However, comparative evidence in minimally invasive cardiac surgery remains limited. This setting poses specific challenges, particularly systemic anticoagulation during cardiopulmonary bypass (CPB), which may restrict the use of neuraxial or paravertebral techniques because of the potential risk of bleeding complications [16–18].

Therefore, identifying analgesic strategies that provide effective pain control while minimizing opioid exposure and maintaining a favorable safety profile remains a clinical priority [19–22]. The BRILMA (block of the lateral branches of the intercostal nerves in the mid-axillary line) targets the lateral cutaneous branches of the intercostal nerves and may reduce procedure-related complications such as pneumothorax compared with conventional intercostal blocks [23]. Cryoanalgesia has demonstrated prolonged postoperative analgesia [24]. To date, no study has directly compared continuous BRILMA and intercostal cryoanalgesia in patients undergoing minimally invasive cardiac surgery via minithoracotomy. Given the specific anatomical and anticoagulation-related constraints of this setting, evidence from thoracic surgery cannot be directly extrapolated to cardiac surgery. Accordingly, this study aimed to compare continuous BRILMA and intercostal cryoanalgesia regarding postoperative pain, opioid consumption, and neuropathic pain after minimally invasive cardiac surgery.

## Methods

### Study design and population

The study was conducted in accordance with the Declaration of Helsinki. Ethical approval was obtained from the Clinical Research Ethics Committee of Vall d’Hebron University Hospital (Barcelona, Spain) (protocol number: PR(AG)324/2022; approved on November 25, 2022). The study was prospectively registered at ClinicalTrials.gov (NCT06086535; October 17, 2023). Written informed consent was obtained from all participants.

All consecutive adult patients (≥ 18 years) undergoing cardiac surgery via minithoracotomy who provided written informed consent were eligible.

Exclusion criteria were: history of chronic or neuropathic pain, regular use of analgesics, neurological disorders with impaired consciousness, pregnancy, and sternotomy approach.

Patients with postoperative catheter displacement or accidental removal were excluded from the final analysis.

Allocation to BRILMA or cryoanalgesia followed routine institutional practice and was based on surgeon preference and device availability. The choice of technique was not based on predefined patient clinical characteristics or anticipated postoperative pain severity.

### Anesthetic and surgical procedure

Standard monitoring included pulse oximetry, electrocardiography, and invasive arterial pressure. Anesthetic induction was performed using fentanyl, midazolam, and a neuromuscular blocking agent (rocuronium or atracurium), followed by endotracheal intubation and placement of a right bronchial blocker to allow selective left lung ventilation. A central venous catheter was inserted via the brachial vein, and venous cannulation for cardiopulmonary bypass was performed through the right internal jugular vein. Transesophageal echocardiography monitoring was used to assess cardiac function and guide vascular cannula placement.

Surgery was performed through a right anterolateral minithoracotomy in the fourth intercostal space. Intraoperative analgesia consisted of fentanyl (total intraoperative dose 15–20 µg/kg).

### Regional analgesic techniques

BRILMA group: The BRILMA was performed by an experienced anesthesiologist at the end of surgery, after thoracotomy closure and before transfer to the cardiac postoperative unit. Patients were positioned supine with slight right lateral tilt, as used during surgery.

Under ultrasound guidance (high-frequency linear probe, in-plane approach), 30 mL of 0.3% ropivacaine were injected in the plane between the serratus anterior and external intercostal muscles at the fifth, fourth, and third intercostal spaces (10 mL per level), proceeding in a caudal-to-cranial direction and corresponding to the surgical field.

A single 19G catheter (StimuLong Sono II NanoLine^®^, Pajunk GmbH, Germany) was inserted at the third intercostal space and advanced within the interfascial plane to allow spread across levels. It was connected to a 250-mL elastomeric pump (DOSI-FUSER^®^, Leventon, Spain) delivering 0.3% ropivacaine at 7 mL/h for 72 h and removed on postoperative day 3.

Cryoanalgesia group: Cryoanalgesia was performed by the surgeon after cardiopulmonary bypass and before thoracotomy closure using a CryoICE^®^ probe (AtriCure^®^, Mason, OH, USA) at − 70 °C. The probe was applied for 2 min to the inferior margin of the third, fourth, and fifth ribs within the surgical field of the right lateral minithoracotomy. This approach targets the intercostal nerves at the corresponding thoracotomy levels.

### Assessments and variables

Pain was assessed at rest and during deep inspiration using the Numerical Rating Scale (NRS) (0–10) after extubation, at 24, 48, and 72 h, at discharge, and at 30 days. For inspiratory assessment, patients performed maximal voluntary inspiration following standardized verbal instructions. Pain was recorded at peak inspiration. NRS scores were categorized as no pain (0), mild (1–3), moderate (4–6), and severe (7–10). Assessments performed during the first 72 postoperative hours were not blinded to treatment allocation.

During hospitalization, all patients received standardized multimodal analgesia: intravenous metamizole (1 g) and paracetamol (1 g) alternated every 4 h. Dexketoprofen (50 mg IV) was administered for NRS 4–6. Tramadol (50 mg IV) was used as rescue therapy. For NRS > 6, morphine (1–2 mg IV boluses) was administered and tramadol discontinued. PCA morphine was initiated in patients with persistent severe pain despite adequate titration with intravenous morphine. It was delivered without basal infusion, with a bolus dose of 1 mg.

At discharge, oral metamizole (1 g) and paracetamol (1 g) were prescribed, with dexketoprofen (25 mg) as rescue.

Neuropathic pain was evaluated using the Douleur Neuropathique 4 (DN4) questionnaire, a previously published and validated instrument [25], at discharge and at 30 days (telephone follow-up). A score ≥ 4 indicated neuropathic pain. Assessments at discharge and at 30 days were performed by investigators blinded to group allocation.

In addition, clinical variables were collected preoperatively, intraoperatively (type of surgery, duration, CPB time, analgesic technique, drains), and postoperatively (time to extubation, need for mechanical ventilation, ICU stay, hospital stay, and pulmonary complications: pneumonia (antibiotics plus fever, opacities, or leukocytosis > 12,000/µL); atelectasis (opacification with shift); and other complications, including Acute Respiratory Distress Syndrome (ARDS) (PaO₂/FiO₂ ratio < 200 with bilateral infiltrates) and pneumothorax (air in the pleural space).

Procedure-related complications, including hematoma, local anesthetic systemic toxicity, and clinically evident neural injury, were also recorded.

### Objectives

The primary objective was to compare postoperative pain at rest over the first 72 h between techniques.

Secondary objectives included pain during inspiration, opioid consumption within 72 h, neuropathic pain incidence, pulmonary complications, and hospital stay.

### Sample size

Sample size was calculated for the primary outcome using postoperative NRS pain scores as the primary measure (Δ = 1.5 NRS points, SD = 2.0, α = 0.05, power = 80%), requiring 28 patients per group.

### Statistical analysis

Categorical variables were summarized as frequencies and percentages and compared using Chi-square or Fisher’s exact tests, as appropriate. Continuous variables were summarized as mean ± standard deviation or median (IQR), according to their distribution. Between-group comparisons for continuous variables were performed using Student’s t test when normally distributed and Mann–Whitney U test when non-normally distributed.

Pain over time was analyzed using linear mixed models with robust standard errors, adjusted for age, sex, and surgical time. These covariates were selected a priori because of their potential influence on postoperative pain perception and analgesic requirements. Estimated marginal means with 95% confidence intervals were reported.

Changes in pain categories over time (NRS < 4 vs. ≥4) were assessed using McNemar’s test. Repeated measures of rescue analgesic requirements, expressed as the number of analgesics required over time, were analyzed using generalized linear mixed models with a Poisson distribution and log-link function. Bonferroni correction was applied for multiple comparisons. Statistical analyses were performed using IBM SPSS Statistics version 30.0. A two-sided p-value < 0.05 was considered statistically significant.

## Results

Sixty-five patients were screened; two were excluded due to postoperative catheter displacement. The final cohort included 63 patients (cryoanalgesia *n* = 30; BRILMA *n* = 33). Mean age was 58.9 ± 14.5 years; 47.8% were women. Two patients in the BRILMA group remained intubated at 24 h and were therefore not evaluable at that time point. Baseline, intraoperative, and postoperative characteristics did not differ between groups (Table 1).

**Table 1:** Preoperative, intraoperative, and postoperative variables of BRILMA and cryoanalgesia techniques

Variable	Cryo (*n* = 30)	BRILMA (*n* = 33)	*p*-value
Preoperative variables			
Age (years)	58.5 ± 16.4	59.2 ± 12.8	0.84
Sex (women/men)	15 (50)/15 (50)	15 (45.5)/18 (54.5)	0.71
Hypertension	9 (30.0)	6 (18.2)	0.27
Diabetes mellitus (type 1/type 2)	1 (3.3)/3 (10)	0 (0)/2 (6.1)	0.33
Renal dysfunction	0 (0)	1 (3.0)	0.51
Neurologic dysfunction (stroke/TIA)	1 (3.3)/1 (3.3)	0 (0)/2 (6.1)	0.17
Pulmonary dysfunction	2 (6.7)	6 (18.2)	0.17
Intraoperative variables			
Type of surgery (MVR/MVr/ASD/TR)	7 (23.3)/16 (3.3)/5 (16.7)/2 (6.7)	9 (27.3)/18 (54.5)/4 (12.1)/2 (6.1)	0.95
CPB time (min)	112.5 ± 42.0	109.8 ± 30.5	0.77
Surgery time (min)	233.0 ± 60.7	238.3 ± 43.4	0.50
Retrocardiac drain	29 (96.7)	33 (100)	0.29
Postoperative variables			
Time to extubation (min)	338.8 ± 235.3	381.6 ± 344.6	0.44
Pulmonary complications (pneumonia/atelectasis/other)	1 (3.3)/1 (3.3)/2 (6.7)	2 (6.1)/0 (0)/2 (6.1)	0.71
IMV requirement	1 (3.3)	3 (9.1)	0.34
NIMV requirement	4 (13.3)	8 (24.2)	0.27
Hospital stay (days)	10.0 ± 5.0	9.6 ± 4.7	0.89
ICU stay (days)	3.0 ± 3.0	2.6 ± 1.0	0.76

Values are expressed as number (percentage) or mean ± SD

Abbreviations: *TIA *transient ischemic attack, *MVR *mitral valve replacement, *MVr *mitral valve repair, *ASD *atrial septal defect, *TR *tumor resection, *CPB *cardiopulmonary bypass, *IMV *invasive mechanical ventilation, *NIM *non-invasive mechanical ventilation

### Pain assessment and analgesic requirements

**Fig. 1:**
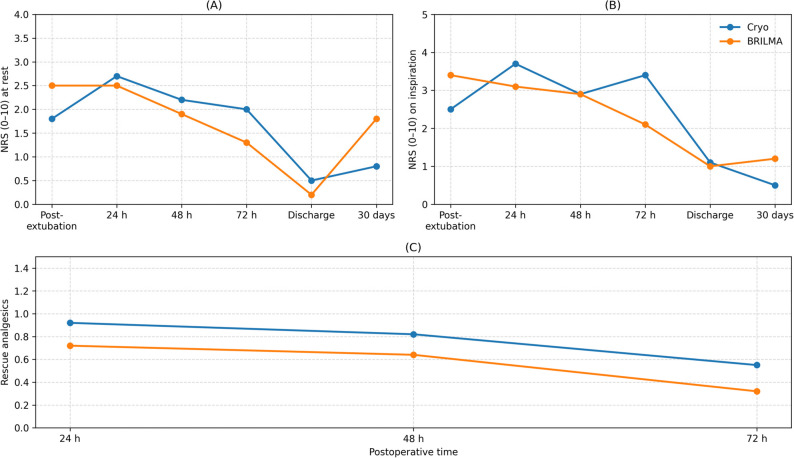
Evolution of pain at rest and during deep inspiration (NRS) and rescue analgesics requirements according to study group. **A** Pain at rest decreased significantly over time in both groups, with different temporal patterns between techniques (group × time interaction *p* = 0.002). **B** Pain during deep inspiration also decreased significantly over time, with significant differences in temporal evolution between techniques (group × time interaction *p* < 0.001). **C** Rescue analgesic requirements decreased significantly during the first 72 postoperative hours (*p* = 0.002), with no significant differences in temporal pattern between groups (*p* = 0.735). Data are presented as estimated marginal means derived from mixed-effects models

Pain decreased significantly over time in both groups during the first 72 postoperative hours, with a significant group-by-time interaction. At rest, BRILMA was associated with lower pain scores during the first 72 h, whereas cryoanalgesia was associated with lower pain scores at 30 days (Fig. 1a; Table 2). During deep inspiration, a similar temporal pattern was observed, with BRILMA showing lower pain scores at 72 h and cryoanalgesia showing lower values at 30-day follow-up (Fig. 1b; Table 2).

**Table 2:** Postoperative pain progression (NRS) at rest and during inspiration: BRILMA vs. cryoanalgesia (linear mixed models)

		Total	Cryo	BRILMA
After extubation	*R*	2.1 (1.7, 2.6)	1.8 (1.1, 2.5)	2.4 (1.8, 3)
	I	3 (2.6, 3.4)	2.6 (2, 3.2)	3.4 (2.9, 4.0)
24 h	R	2.6 (2.2, 3.0)	2.7 (2.1, 3.3)	2.5 (1.9, 3.1)
	I	3.5 (3.1, 3.9)	3.7 (3.1, 4.3)	3.2 (2.6, 3.8)
48 h	R	2.0 (1.6, 2.3)	2.2 (1.7, 2.7)	1.8 (1.3, 2.3)
	I	2.9 (2.5, 3.3)	3.0 (2.4, 3.6)	2.8 (2.3, 3.4)
72 h	R	1.5 (1.1, 1.9)	2.0 (1.3, 2.7)	1.0 (0.5, 1.4)
	I	2.8 (2.4, 3.2)	3.5 (2.9, 4.1)	2.1 (1.6, 2.7)
At discharge	R	0.3 (0.1, 0.5)	0.5 (0.1, 0.8)	0.2 (-0.02, 0.4)
	I	1.1 (0.7, 1.5)	1.2 (0.6, 1.8)	1.0 (0.5, 1.6)
At 30 days	R	1.3 (0.9, 1.8)	0.8 (0.3, 1.3)	1.8 (1.1, 2.6)
	I	0.9 (0.5, 1.3)	0.5 (-0.1, 1.1)	1.2 (0.7, 1.8)
Δ discharge–extubation	R	-1.8 (-2.3, -1.3)***	-1.3 (-2.1, -0.5)***	-2.2 (-2.8, -1.6)***
	I	-1.9 (-2.4, -1.4)***	-1.4 (-2.1, -0.7)***	-2.4 (-3.1, -1.7)***
Δ 30 days-discharge	R	1.0 (0.5, 1.4)***	0.3 (-0.2, 0.9)	1.6 (0.9, 2.3)***
	I	-0.2 (-0.7, 0.2)	-0.7 (-1.4, 0.0)	0.18 (-0.5, 0.9)
Δ 30 days-extubation	R	-0.8 (-1.4, -0.2)*	-1.0 (-1.9, -0.2)*	-0.6 (-1.5, 0.2)
	I	-2.1 (-2.6, -1.6)***	-2.0 (-2.7, -1.3)***	-2.2 (-2.9, -1.5)***
Pain evolution, *p*-valor	R	F(5,360) = 29.24, *p* < 0.001	Group per time interaction	F(5,360) = 3.89, *p* 0.002
	I	F(5,303) = 39.09, *p* < 0.001	Group per time interaction	F(5,303) = 5.45, *p* < 0.001

Abbreviations: R, at rest; I, deep inspiration; Δ, difference. Values are expressed as estimated marginal means (95% CI). Pain was assessed using the Numerical Rating Scale (NRS, 0–10) at rest and during deep inspiration after extubation, at 24, 48, and 72 h, at hospital discharge, and at 30 days. Linear mixed models were adjusted for age, sex, and surgery duration. Group × time interaction analyses are shown in the final rows of the table. Clinically relevant between-group differences were mainly observed at 72 h and at 30 days, where BRILMA was associated with lower early postoperative pain, whereas cryoanalgesia showed lower late pain scores. *p < 0.05, **p < 0.01, ***p < 0.001

### Pain intensity categories

At 72 h, moderate-to-severe pain (NRS ≥ 4) at rest was less frequent in the BRILMA group but persisted in approximately one quarter of cryoanalgesia patients (Table 3). A similar pattern was observed during deep inspiration. At discharge, pain improved in both groups. At 30 days, the proportion of patients with moderate pain increased in the BRILMA group, whereas it remained low in the cryoanalgesia group (Table 3).

**Table 3:** Postoperative NRS pain intensity categories at rest and during deep inspiration according to analgesic technique from extubation to 30 days

Time point	Pain category	Cryo (*R*)	Cryo (I)	BRILMA (*R*)	BRILMA (I)	*p*-value (*R*)	*p*-value (I)
After extubation	Mild (0–3)	25 (83.3)	23 (76.7)	26 (78.8)	22 (66.7)	0.85	0.67
	Moderate (4–6)	4 (13.3)	5 (16.7)	5 (15.2)	8 (24.2)		
	Severe (7–10)	1 (3.3)	2 (6.7)	2 (6.1)	3 (9.1)		
24 h	Mild (0–3)	22 (73.3)	17 (56.7)	24 (77.4)	21 (67.7)	0.91	0.62
	Moderate (4–6)	5 (16.7)	10 (33.3)	4 (12.9)	7 (22.6)		
	Severe (7–10)	3 (10.0)	3 (10.0)	3 (9.7)	3 (9.7)		
48 h	Mild (0–3)	23 (76.7)	20 (66.7)	28 (84.8)	26 (78.8)	0.48	0.34
	Moderate (4–6)	6 (20.0)	9 (30.0)	5 (15.2)	5 (15.2)		
	Severe (7–10)	1 (3.3)	1 (3.3)	0 (0)	2 (6.1)		
72 h	Mild (0–3)	21 (70.0)	18 (60.0)	32 (97.0)	29 (87.9)	0.02	0.01
	Moderate (4–6)	8 (26.6)	7 (23.3)	1 (3.0)	4 (12.1)		
	Severe (7–10)	1 (3.3)	5 (16.7)	0 (0)	0 (0)		
Discharge	Mild (0–3)	29 (96.7)	29 (96.7)	33 (100)	33 (100)	0.29	0.29
	Moderate (4–6)	1 (3.3)	1 (3.3)	0 (0)	0 (0)		
30 days	Mild (0–3)	28 (93.3)	29 (96.7)	25 (75.8)	27 (81.8)	0.05	0.16
	Moderate (4–6)	2 (6.7)	1 (3.3)	8 (24.2)	5 (15.2)		
	Severe (7–10)	0 (0)	0 (0)	0 (0)	1 (3.0)		

Values are expressed as number (%). Postoperative pain intensity categories were defined according to the Numerical Rating Scale (NRS) as mild (0–3), moderate (4–6), and severe (7–10). *p* values correspond to between-group comparisons at each postoperative time point. Abbreviations: *R *at rest, *I *deep inspiration, *NRS *Numerical Rating Scale

**Fig. 2:**
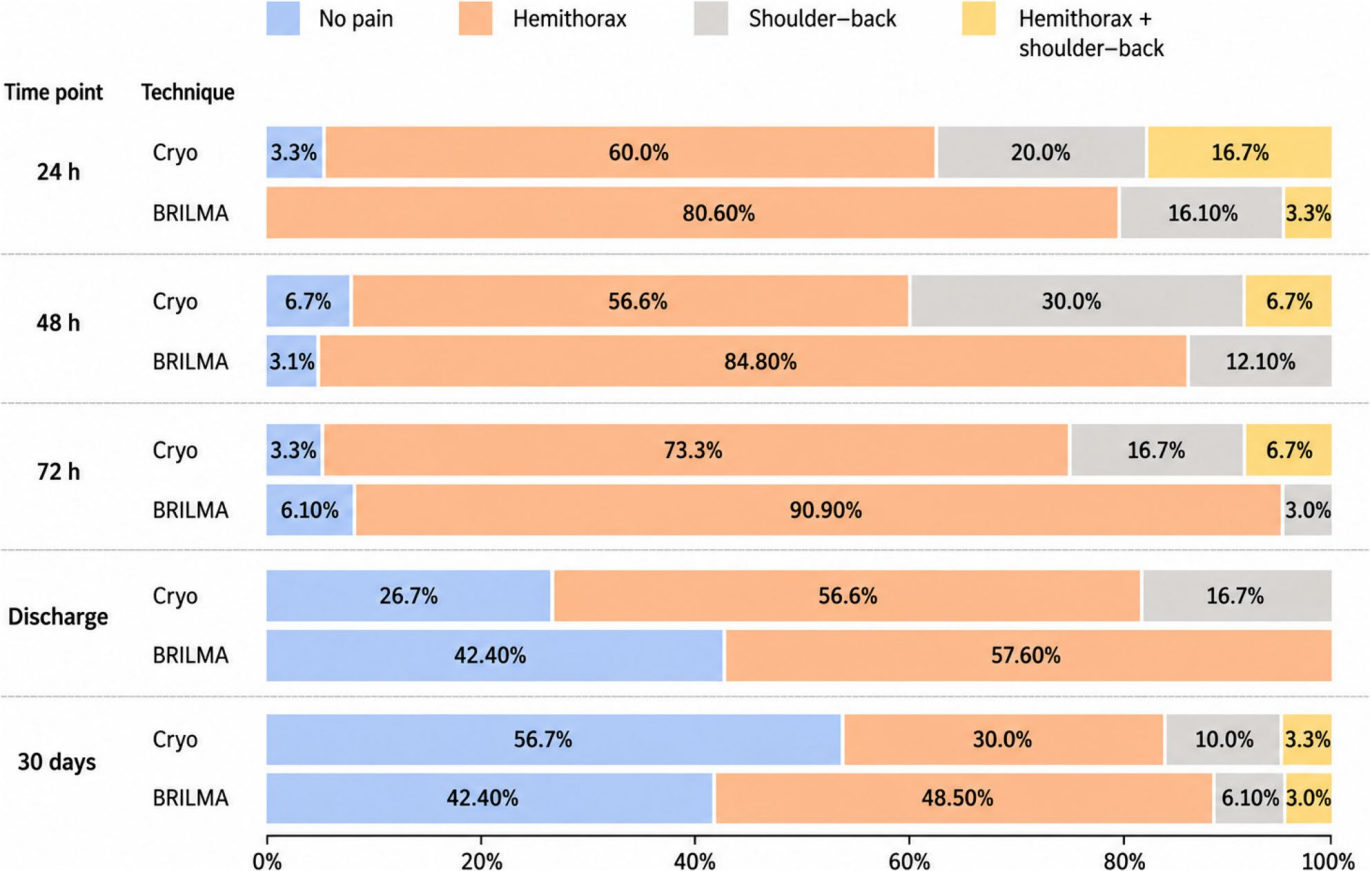
Pain location over time according to study group (C: cryoanalgesia, B: BRILMA) at different postoperative time points

Within-group analysis using McNemar’s test showed significant reductions in moderate-to-severe pain in the cryoanalgesia group between 72 h and discharge (rest *p* = 0.016; inspiration *p* < 0.001). In the BRILMA group, moderate-to-severe pain was already infrequent at 72 h and remained low thereafter. Pain location varied over time, with distinct patterns between techniques (Fig. 2).

### Analgesic consumption

Repeated-measures analysis of rescue analgesic requirements, expressed as the number of analgesics required over time, showed a significant reduction during the first 72 postoperative hours (*p* = 0.002), with no significant differences in temporal pattern between groups (*p* = 0.735; Fig. 1c). Overall opioid exposure was low in both groups. The median total morphine dose, including PCA administration, was 0 mg (IQR 0–6) in the cryoanalgesia group and 0 mg (IQR 0–0) in the BRILMA group (*p* = 0.068). When expressed as intravenous morphine equivalents, total opioid consumption was 5 mg (IQR 0–12.25) versus 0 mg (IQR 0–5), respectively (*p* = 0.067).

A greater proportion of patients in the cryoanalgesia group required morphine boluses and PCA during the early postoperative period (Fig. 1c). Specifically, PCA morphine was required in 9 (30.0%) cryoanalgesia patients compared with 4 (12.1%) BRILMA patients (*p* = 0.08). Tramadol (*p* = 0.238) and dexketoprofen (*p* = 0.571) requirements did not differ significantly between groups.

### Neuropathic pain

**Fig. 3:**
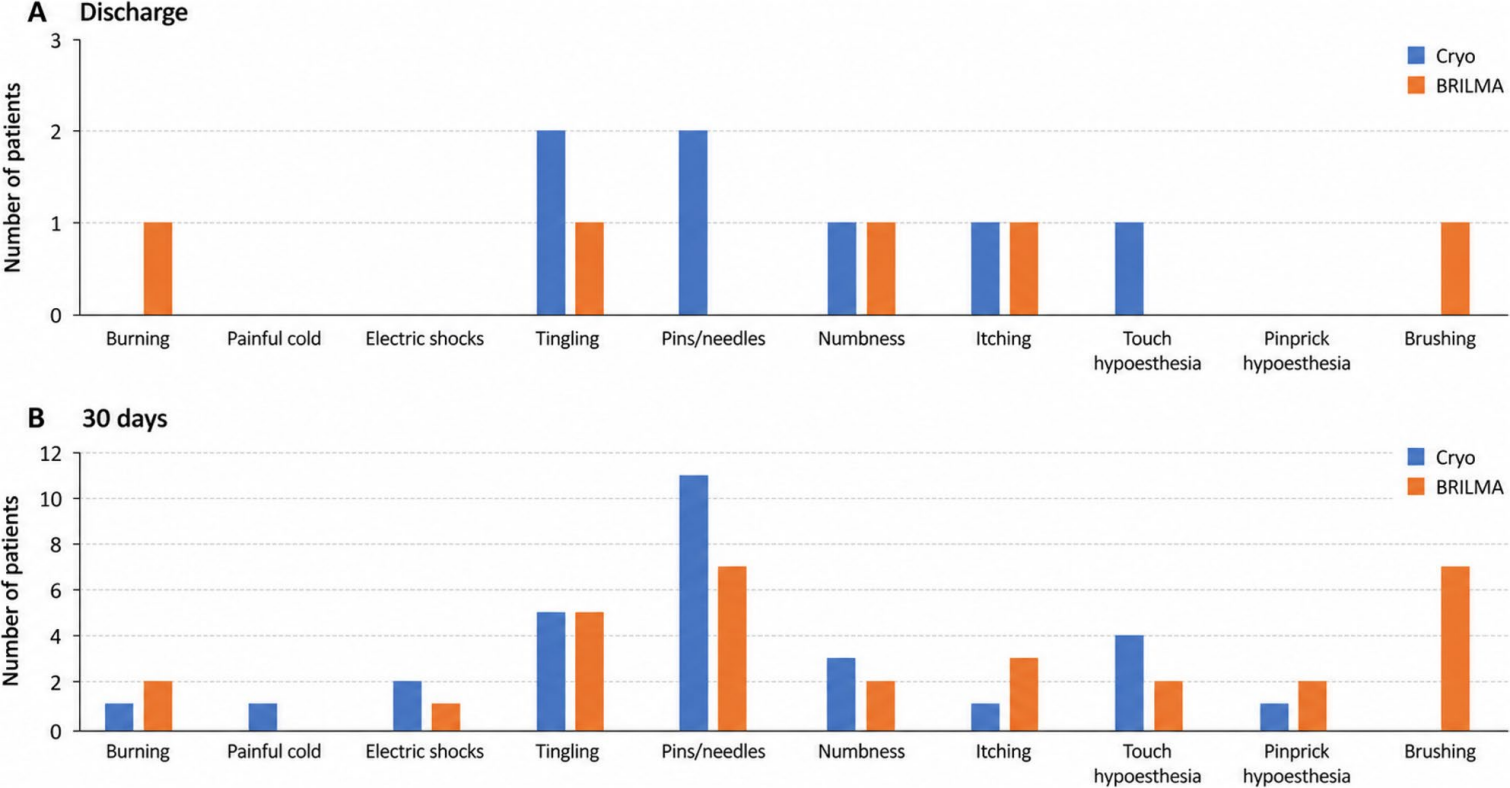
Number of patients with neuropathic pain at discharge (A) and at 30 days (B) according to study group (cryoanalgesia and BRILMA). Neuropathic pain was defined as a DN4 score ≥ 4

Neuropathic pain was uncommon. At discharge, 2 patients (3.2%) had DN-4 ≥ 4; at 30 days, 6 patients (9.5%) met criteria, with similar distribution between groups (Fig. 3). In the cryoanalgesia group, three patients (10%) developed new-onset neuropathic pain between discharge and follow-up, whereas the patient with neuropathic pain at discharge no longer met DN4 criteria at 30 days. In the BRILMA group, two patients (6%) developed neuropathic pain at 30 days, whereas the patient with neuropathic pain at discharge remained symptomatic at follow-up. No procedure-related complications such as hematoma, local anesthetic systemic toxicity, or clinically evident neural injury were observed in either group during hospitalization or follow-up.

## Discussion

The main finding of this study is that both intercostal cryoanalgesia and continuous BRILMA were associated with effective postoperative pain control after minimally invasive cardiac surgery, with distinct temporal profiles. BRILMA was associated with lower early postoperative pain scores during the first 72 h, whereas cryoanalgesia showed greater variability within the first 72 h but tended to maintain lower pain levels at 30 days. Overall opioid exposure was low and did not significantly differ between techniques, and neuropathic pain incidence was similarly low, suggesting no major differences in short-term safety outcomes within the limitations of this study. These results suggest that the choice of regional analgesia may be tailored according to the expected temporal course of pain and clinical priorities, as no relevant differences were observed in postoperative pulmonary complications or hospital length of stay.

Consistent with Dokollari et al. [26] and Lau et al. [27], our results suggest that cryoanalgesia may provide a gradually established analgesic effect extending into the subacute period. However, unlike the FROST study [27], which reported sustained reductions in pain, we observed a mild rebound in resting pain at one month, although still within clinically acceptable levels. This may reflect early nerve regeneration or differences in post-discharge analgesic management. In line with Weksler et al. [12], cryoanalgesia did not provide additional benefit over alternative regional techniques during the early postoperative period.

Regarding BRILMA, prior evidence in cardiac thoracotomy is lacking. Our findings suggest effective and sustained analgesia throughout hospitalization, with minimal opioid requirements, and lower early pain scores compared with cryoanalgesia during the first 72 h, likely reflecting the latency of cryoanalgesia during its stabilization phase. This temporal difference may reflect distinct mechanisms of action between both techniques. Continuous BRILMA may provide earlier neural blockade through local anesthetic infusion and may therefore be more suitable for acute postoperative pain control during the first postoperative days. In contrast, cryoanalgesia may induce a delayed interruption of nerve conduction, which may explain the less pronounced analgesic effect during the acute phase but the trend toward lower pain levels at 30 days. Consequently, both techniques may target different phases of postoperative pain evolution rather than representing directly interchangeable strategies. Compared with single-injection BRILMA studies [23, 28] continuous infusion may extend analgesic duration. Similarly, Yao et al. [29] reported that catheter-based continuous intercostal techniques improved early pain control. Although intercostal blocks are effective in thoracic surgery [30], limitations compared with neuraxial techniques have been described [31]. BRILMA acts via an interfascial plane targeting the lateral cutaneous branches of the intercostal nerves, potentially reducing procedural risks while preserving analgesic efficacy.

In our study, cryoanalgesia was associated with higher early inspiratory pain peaks but lower pain at 30 days, consistent with a delayed yet sustained analgesic effect [11]. Previous thoracic surgery studies have suggested that cryoanalgesia may not provide greater benefit than epidural analgesia for movement-related pain [32]. In contrast, BRILMA was associated with earlier and more stable control of dynamic pain. At 30 days, both groups showed low inspiratory pain and a slight predominance of resting discomfort, which has been attributed by some authors to resting hypervigilance [33].

Together, these findings raise the hypothesis that combining BRILMA (rapid onset, early control) with cryoanalgesia (delayed but prolonged effect) could provide broader analgesic coverage across acute and subacute phases in selected high-risk patients. However, the present study was designed to compare both techniques as independent analgesic strategies routinely used in our institution, and no combined-treatment group was included. Future studies may explore whether combining interfascial blockade with cryoanalgesia could provide broader analgesic coverage in selected patients.

Pain was predominantly localized to the surgical hemithorax during the first 48–72 h, with some radiation to the shoulder–back region, potentially related to intraoperative positioning and musculoskeletal strain. Shoulder–back pain was more frequent in the cryoanalgesia group, possibly because cryoanalgesia selectively targets intercostal nerves and may not address deeper musculoskeletal sources. By contrast, BRILMA may also contribute to attenuation of referred dorsal pain through local anesthetic spread into deeper fascial planes and anti-inflammatory effects [34]. These findings are consistent with Lau et al. [27], where cryoanalgesia was associated with greater pain at specific postoperative time points.

A greater proportion of cryoanalgesia patients required morphine boluses and morphine PCA, although between-group differences in cumulative opioid exposure did not reach statistical significance. This may reflect the low overall opioid requirements achieved with the standardized multimodal regimen. Notably, some BRILMA patients showed a mild pain rebound after catheter removal, suggesting the importance of effective transition analgesia. Compared with the FROST trial [27], our opioid findings showed an opposite direction, which may relate to methodological differences: FROST used less standardized systemic analgesia without a clearly specified rescue protocol, whereas both groups in our study followed the same multimodal regimen, differing only in regional technique. This supports the interpretation that the observed differences may have been associated with the regional technique.

Neuropathic pain at 30 days was low and similar between groups, which compares favorably with previous reports of chronic post-thoracotomy pain and neuropathic components [35]. Regional techniques may reduce neuropathic features compared with systemic analgesia [36, 37]. Evidence for cryoanalgesia remains mixed: some studies suggest increased neurosensory symptoms during the early weeks, potentially related to nerve regeneration [12]. In minimally invasive cardiac surgery, data remain scarce; FROST [27] and Dokollari et al. [26] reported low neurosensory symptoms after cryoanalgesia but did not apply validated diagnostic instruments, limiting comparability. In contrast, we used the DN-4 scale, providing structured assessment. Histological studies suggest that cryoanalgesia induces axonal degeneration with preservation of the perineurium, allowing orderly nerve regeneration without neuroma formation [38]. Overall, both techniques were associated with a low incidence of neuropatic pain in this setting.

### Limitations of the study

This was a single-center prospective comparative observational study. Treatment allocation was not randomized, which may introduce residual confounding despite comparable baseline characteristics and multivariable adjustment. Therefore, causal inferences should be interpreted with caution. However, this design reflects real-world clinical practice and provides clinically relevant comparative data in a setting where evidence remains limited. To our knowledge, no prior studies have directly compared continuous BRILMA and intercostal cryoanalgesia in minimally invasive cardiac surgery, and available data are mainly derived from thoracic surgery, which differs in surgical approach, pain mechanisms, and patient population.

The sample size was calculated for the primary outcome and was not specifically powered for secondary outcomes such as opioid consumption or less frequent events, including neuropathic pain.

Follow-up was limited to 30 days, precluding assessment of long-term chronic pain and late neurosensory outcomes. This may be particularly relevant for cryoanalgesia, whose potential benefits could extend beyond the early postoperative period.

Finally, although pain assessments followed a standardized protocol, blinding was limited to neuropathic pain evaluations at discharge and at 30 days. In-hospital postoperative pain assessments were not blinded and may therefore be subject to measurement and performance bias.

## Conclusion

This comparative study suggests that both intraoperative cryoanalgesia and continuous BRILMA are associated with effective postoperative analgesia after minimally invasive cardiac surgery, with distinct temporal profiles. BRILMA tended to be associated with lower early pain scores during the first 72 h, particularly at rest, whereas cryoanalgesia showed a more gradual onset with comparatively lower pain levels at 30 days. Overall opioid exposure and neuropathic pain incidence were low and comparable between techniques. These findings suggest that the selection of regional analgesic strategy may be individualized according to the expected temporal pattern of postoperative pain, depending on whether early postoperative analgesia or sustained subacute pain control is prioritized.

## Data Availability

The datasets used and analyzed during the current study are available from the corresponding author on reasonable request.

## Abbreviations

ASD: Atrial Septal Defect
BRILMA: Block of the Lateral Branches of the Intercostal nerves in the Mid-Axillary line
CPB: Cardiopulmonary Bypass
C: Cryoanalgesia
DN4: Douleur Neuropathique 4
IMV: Invasive Mechanical Ventilation
I: Inspiration
MICS: Minimally Invasive Cardiac Surgery
MVR: Mitral Valve Replacement
NIMV: Non-Invasive Mechanical Ventilation
MVr: Mitral Valve Repair
NRS: Numerical Rating Scale
R: at Rest
TIA: Transient Ischemic Attack
TR: Tumor Resection
